# Developmental Coordination Disorder: The Importance of Grounded Assessments and Interventions

**DOI:** 10.3389/fpsyg.2018.02409

**Published:** 2018-12-04

**Authors:** Mats Niklasson, Peder Rasmussen, Irene Niklasson, Torsten Norlander

**Affiliations:** ^1^Center for Research and Development, Evidens University College, Göteborg, Sweden; ^2^Center for Sensorimotor Research, Vestibularis Clinic, Kalmar, Sweden; ^3^Department of Neuroscience and Physiology, Institute of Child and Adolescent Psychiatry, Sahlgrenska University Hospital, Göteborg, Sweden

**Keywords:** DCD, primary reflexes, sensorimotor disorders, sensorimotor therapy, vestibular dysfunction, kinesthetic-vestibular developmental model, arrested development, perceptual priming

## Abstract

This focused review is based on earlier studies which have shown that both children and adults diagnosed as having developmental coordination disorder (DCD), benefited from sensorimotor therapy according to the method Retraining for Balance (RB). Different approaches and assessments for children and adults in regard to DCD are scrutinized and discussed in comparison to RB which mainly includes (a) vestibular assessment and stimulation (b) assessment and integration of aberrant primary reflexes and (c) assessment and stimulation of auditory and visual perception. Earlier results indicate that the process of Sensorimotor therapy using RB techniques could be described according to a conceptual Kinesthetic-Vestibular Developmental Model (KVDM) whereby the training elicited temporary physical and psychological regressions followed by transformations i.e., positive physical and psychological development. We have also seen that this recurring pattern is similar for children and adults. In our conceptual model vestibular stimulation (perceptual priming) stimulates the nervous system, which might enhance object-related priming. This perceptual priming will also assist the suppression of persistent aberrant primary reflexes. In order to develop effective methods for assessment and intervention of DCD over the life span the importance of primary reflex inhibition and vestibular stimulation as well as a combination of bottom-up and top-down approaches have to be considered.

## Introduction

This Focused review aim at not only following-up our recent empirical study (Niklasson et al., 2015) but also at filling a gap in the literature concerning assessments and interventions of **developmental coordination disorder (DCD)**. So far a grounded approach i.e., an approach, which has its starting point in fundamental neurological development including aberrant **primary reflex** assessment and inhibition as well as vestibular assessment and stimulation has been missing. We aim at filling that gap.

KEY CONCEPT 1 **Developmental Coordination Disorder (DCD)**

Characterized by a delayed and immature gross and fine motor development without obvious intellectual or medical causes, DCD is defined as a neurodevelopmental motor disorder.

KEY CONCEPT 2 **Primary reflexes**

Primary reflexes are movement patterns, which are complex, stereotyped and automatic. They are present at birth but are supposed to be integrated as the nervous system matures during the first year of life.

Our recent study (Niklasson et al., 2015) confirmed that motor problems don't disappear with age and showed that it was possible to use both the same diagnostic instrument and treatment method for children, adolescents and adults diagnosed with Developmental Coordination Disorder (DCD). The study also concluded that sensorimotor problems in childhood should be taken seriously. The main purpose of this Focused review is to put our recent study in a wider perspective. We also intend to give a more thorough description of both the method Retraining for Balance (RB) and **Sensorimotor therapy (SMT)**.

KEY CONCEPT 3 **Sensorimotor therapy (SMT)**

SMT is a process-oriented approach to sensorimotor training which emphasize the interdependence between primary reflex suppression and vestibular stimulation.

We start with a short presentation describing the present status of DCD followed by a description of three approaches to intervention. There after we discuss frequently used assessment instruments aimed for children and for adults. Finally a presentation of RB will follow and we close by discussing possible advantages for the use SMT for both younger and older children as well as for adults.

## The Present Status of DCD

So far no “gold standards” for assessments and interventions of DCD have been established although several approaches have been used (Dewey et al., 2011; Smits-Engelsman et al., 2012). Neither are there any instruments for assessment of DCD to be used over the lifespan, from childhood to adulthood (Kirby and Sugden, 2007). DCD is still recognized as a “hidden problem” (Caçola, 2016) and “currently among the most neglected problems in the whole field of developmental medicine/child neuropsychiatry” (Gillberg, 2017).

During the last century children with clumsy movements and motor coordination difficulties have been described and often differently labeled in both medical and psychological literature (Ahonen et al., 2004; Tupper and Sondell, 2004), but since 1994 the construct of Developmental Coordination Disorder (DCD) (American Psychiatric Association., 1994, 2013) has been preferred. Although motor problems have been well known and described for long and despite an estimated prevalence of between 6 and 13% among all school-aged children (Smits-Engelsman et al., 2012) no consensus regarding symptoms, etiology (Gomez and Sirigu, 2015; Vaivre-Douret et al., 2016), terminology and concepts (Peters et al., 2001; Wilson, 2004; Gibbs et al., 2007) have been reached. There are only recommendations (Blank et al., 2012).

With extended knowledge about DCD and its persistence throughout life as well as knowledge about its different co-morbidities (Rasmussen and Gillberg, 2000), research regarding detection (Mahoney et al., 2004), assessment instruments (Schoemaker and Wilson, 2015), interventions in early childhood (Wilson, 2004), and in schools (Norlander et al., 2005) ought to be priority not the least due to a high risk of additional problems such as depression and anxiety (Caçola, 2016). Another problem tending to remain into adulthood (Tal-Saban et al., 2012; Purcell et al., 2015) is a sedentary or physical inactive behavior. In a qualitative study, one of us used a phenomenological perspective (Bergman and Norlander, 2005) in order to understand participant's physical inactivity. It was concluded that the resistance to physical activity might be due to an “unidentified psychological barrier.” Participant's had a desire to leave their inactive life but seemed to be stuck in an unbreakable vicious circle. This leaves us with the question of which approaches would be most suitable for intervention.

## Approaches

Presently, approaches to DCD interventions belong mainly to either deficit-/process-oriented approaches (bottom-up) or to functional skills/task-oriented approaches (top-down, dynamic systems theory). Although these approaches have their weaknesses and strengths it would be useful to combine them when it comes to pediatric motor skills assessment (Kennedy et al., 2013).

### Bottom-up

A deficit- or process-oriented approach to intervention (Wilson, 2004; Sugden, 2007; Blank et al., 2012; Smits-Engelsman et al., 2012) also known as the General Abilities Approach (Pless and Carlsson, 2000) is based on maturational, neurodevelopmental theory and on the assumption that underlying sensorimotor processes need to be remedied and improvements lead to a better performance of motor skills. An example of a bottom-up approach is Sensory Integration therapy (SIT) (Ayers, 1972). SIT uses sensory stimulation in order to enhance motor development and cognitive abilities but does not include integration of primary reflexes in order to develop motor milestones such as rolling, belly crawling and crawling on hands and knees (Niklasson, 2013a; Lin et al., 2014).

### Top-down

A functional skills/task-oriented approach to intervention (Pless and Carlsson, 2000; Wilson, 2004; Thelen and Smith, 2006; Sugden, 2007; Blank et al., 2012; Smits-Engelsman et al., 2012) is a top-down approach, which implies that the child or adult is taught how to handle a task, a special skill or a strategy in its environmental context. The most promising and also the most recommended method is Cognitive Orientation to Daily Occupational Performance (CO-OP). Coming from the field of occupational therapy, CO-OP is mainly focused on cognitive strategies for the acquisition of age-specific functional skills.

### Dynamical Systems Theory

Among skills/task-oriented approaches a perspective on motor development provided by the framework of dynamical systems theory (DST) has gained much attention and has been influential for developmental psychology research for more than 25 years, mainly due to its connection to physical science (Hollenstein, 2011). Originated in mathematics DST emphasizes i.e., “changes over time,” “emergence,” “non-linear,” and “self-organization” (Thelen and Smith, 2006). Using the framework of DST a dynamic systems perspective on motor development (Goldfield and Wolff, 2004) holds that motor behavior is an emerging result of the interaction between different subsystems. Without any special entity directing it, the system is self-organizing with changes appearing to be non-linear. An example could be the transition from crawling to walking. According to the dynamic systems perspective this transition is due to a dynamic interaction between the child's ability, the context of the environment and the task in question. An important force for a developmental change is the child's motivation to explore (Thelen, 2000; Thelen and Smith, 2006). According to the dynamic perspective the nervous system is a part of an embodied system with the brain acting as a medium “informationally coupled” to the environment yielding patterns for control and coordination mainly through visual information (Goldfield and Wolff, 2004). Shumway-Cook and Woollacott (1995) meant that one limitation of this perspective could be the anticipation that the nervous system is supposed to play a rather unimportant role.

From our point of view this remark is well founded since our studies (Niklasson et al., 2009, 2015) have indicated that the persistence of aberrant primary reflexes together with a vestibular dysfunction are hindrances for the development of motor milestones. Therefore, we tentatively suggest that motivation is not enough. The child must primarily be able to handle gravity (Adolph and Franchak, 2017) in order to develop a dynamic interaction between perception and movement.

## Assessments

Approaches to DCD interventions such as those described above are strongly dependent on high quality instruments in order to monitor complex training processes.

According to recent recommendations from the European Academy of Childhood Disability (Blank et al., 2012) a DCD diagnosis not only requires standardized, valid and reliable motor tests and questionnaires but also a clinical examination and history taking. There are several instruments available for the assessment of DCD in children but they are scarce regarding the diagnosis of adults and so far absent (Kirby and Sugden, 2007) concerning instruments to be used over the lifespan, from childhood to adulthood. However, a recent study by Sigmundsson et al. (2016), which evaluated a test battery aimed at determine fine- and gross motor competence across the life span showed promising results.

### Instruments Aimed for Children

#### Sensory and Integration Praxis Tests (*SIPT*)

Sensory and Integration Praxis Tests *(SIPT)* Ayers (1989); Mailloux (1990). SIPT is standardized for children aged between 4 years and 11 years 8 months and has 17 subtests divided into four parts: (1) tactile processing and discrimination, (2) vestibular and proprioceptive processing, (3) bilateral integration, sequencing, and praxis and finally (4) perception of shape, space and visuo-motor coordination. The test score reliability is high when it comes to determining sensory motor integration disorder. However, additional information such as case history and clinical observations needs to be added for reliable interpretations of scores (Jorquera-Cabrera et al., 2017). According to Wilson (2004) the test provides limited information about how the perceptual-motor system works and the validity is low. SIPT has not been revised since 1989, which might be one limitation and another could be the 2 h estimated for administration (Jorquera-Cabrera et al., 2017).

#### Movement ABC-2

*Movement Assessment Battery for Children-second edition* (MABC-2) (Henderson et al., 2007) is standardized for children aged 3 to 16 years and probably one of the most commonly used and recommended tests for assessment of motor impairment. MABC-2 is designed to provide a general index and includes a performance part and a checklist. The performance part, which takes 20–40 min to administer, is descriptive and product oriented and mainly aimed at evaluating movements at the functional level (Wilson, 2004). This part covers tests for ball skills, manual dexterity and static and dynamic balance. However, as stressed by Barnett (2014) it is not a “test of DCD,” rather a general motor test used to detect motor difficulties. The checklist is a validated questionnaire (Wilson et al., 2009; Blank et al., 2012) to be completed by a parent or by another adult, familiar with the child's general motor function. For a proper evaluation it is recommended that both parts of the test are used.

#### BOT−2

*Bruininks-Oseretsky Test of Motor Profiency-2* (BOT-2) (Bruininks and Bruininks, 2005) is another commonly used and recommended test. BOT-2 is standardized for children and adolescents aged 4 to 21 years 11 months and the complete test takes 45–60 min to perform. Like Movement ABC-2 the test is not aimed for diagnostic purposes. It is rather a motor test designed to detect motor difficulties (Barnett, 2014). The test, which generates a general motor ability factor is divided into 8 sections: Fine motor precision, Fine motor integration, Manual dexterity, Upper-limb coordination, Bilateral coordination, Balance, Running speed/agility, and Strength.

It was also mentioned (Kaplan et al., 1998) that while MABC allows instructions only ahead of performance and BOT allows for coaching during the test, MABC might penalize children with difficulties to remember. Furthermore, neither of the tests measures the quality of movement completely.

#### DCDQ′07

*The Developmental Coordination Disorder Questionnaire 2007* (DCDQ′07) (Wilson et al., 1998, 2009; Wilson and Crawford, 2012) is a questionnaire for parents aiming at identifying subtle motor problems in children aged 8 to 14 years 6 months. The questionnaire consists of 15 items divided into three factors: Control during movement, Fine motor and handwriting, and General coordination. Although the DCDQ'07 is a reliable and valid instrument (Montoro et al., 2016) it cannot by itself be used to identify DCD (Pannekoek et al., 2012). A recent study by Montoro et al. (2016) indicated concurrent validity between DCDQ and MABC-2 suggesting the use of MABC-2 as an indicator for DCD.

#### Retraining for Balance

*Retraining for Balance* (RB) (Niklasson and Niklasson, 2007a,b; Niklasson et al., 2007) is an umbrella term for both assessments and interventions used in sensorimotor therapy (SMT). The method mainly includes (a) vestibular assessment and stimulation (b) assessment and integration of aberrant primary reflexes and (c) assessment and stimulation of auditory perception. A more thorough description will follow below.

### Instruments Aimed for Adults

In accordance with substantial research (e.g., Rasmussen and Gillberg, 2000), which has shown that individuals do not grow out of motor problems, DSM-5 (American Psychiatric Association., 2013; Purcell et al., 2015) recognizes DCD as persistent throughout the life span. However, standardized tests for adults are lacking. Sometimes MABC-2 and BOT-2 are used but their psychometric properties when used to assess adults are still unclear (Barnett, 2014). So far, a self-rating questionnaire such as *the Adult Developmental Co-ordination Disorders/Dyspraxia Checklist (ADC)* (Kirby et al., 2010) is most commonly used. ADC is a comprehensive instrument, which was developed and tested on individuals in the age range of 17 to 42 years. It consists of 40 items, 10 rates childhood performance while 30 rates current adult performance. The items cover both motor behavior, for example movement skills and handwriting, and behavior beyond the motor capacity such as social skills and general organization. Despite the growing awareness and knowledge about DCD in adults during recent years, evidence based research is still scarce (Missiuna et al., 2012; Tal-Saban et al., 2012) and the need to develop appropriate assessment tools for adults is urgent (Barnett, 2014).

Our recent study (Niklasson et al., 2015), which used the method *Retraining for Balance* in a group of adults, indicated promising results. The method follows hierarchical principles (Wiest, 2012) and includes both assessment tools and guidelines for intervention regarding persistent primary reflexes and vestibular dysfunction. The main logic behind RB is that un-integrated primary reflexes together with vestibular dysfunction cause an arrested sensorimotor development, which will be released only through a proper and methodical suppression of the reflexes. This is why the method can be used and also apparently works in all age groups. There is a strong connection between certain primary reflexes and the **vestibular system**. One example of special interest is the Moro reflex, which is expected to be gradually suppressed during the first 6 months of life. Nonetheless it was frequently found among participants in both the child- and the adult-group in our study (Niklasson et al., 2015). Another study by Konicarova and Bob (2012) showed that a persisting Moro reflex was closely linked to Attention Deficit Hyperactivity Disorder (ADHD) in school children aged 8 to 11 years.

KEY CONCEPT 4 **The vestibular system**

The vestibular system is the organ for detection of gravity and for movement. A balance system, which regulates i.e., eye-movements and postural reflexes.

In the newborn the Moro reflex is unconditioned and released through a sharp alteration of the infant's position in space. The first form of anxiety is then the fear of falling, closely connected to the excitement of the vestibular part of the eighth cranial nerve. The other part of this cranial nerve, which is connected to the auditory system, is stimulated by sudden loud noises. Since the newborn is rather insensitive to noise the reaction and fear for loud and/or sudden noises, a Startle reflex develops somewhat later and is conditioned. The common denominator for both anxiety and fear is an irritation of at least one of the branches of the eighth cranial nerve (Feldenkrais, 1988). Although it has been argued that The Moro reflex and the Startle reflex are different entities (e.g., Rousseau et al., 2017) they are thought to be part of the same developmental chain (Goddard Blythe, 2014) and therefore we find it likely that they are two sides of the same coin. In a recent study, Poli and Angrilli (2015) demonstrated that a strong Startle reflex was associated with higher anxiety levels. We speculate that retention of the Moro reflex and a too easily elicited Startle reflex among both adolescents and adults with sensorimotor problems and/or anxiety is more common than expected. Therefore, we suggest that the hitherto neglected roles of persistent primary reflexes and of vestibular dysfunction ought to be parts of future DCD diagnosing. Retraining for Balance constitute such a diagnosing instrument.

## The Framework of Retraining for Balance

### Theory

Through a combination of theoretical knowledge (e.g., Ayers, 1972; Thelen, 2000) and clinical practice (e.g., Blythe, 2009) we came to understand the importance of mixing the inhibition of aberrant primary reflexes with vestibular stimulation and thereby creating assessments and interventions grounded in fundamental neurological development. In a previous naturalistic study (Niklasson et al., 2009), comprised of 232 children and adolescents diagnosed with **sensorimotor disorder**, results showed that the integration of persisting primary reflexes together with vestibular stimulation according to the method Retraining for Balance (RB) enhanced sensorimotor development. At the time we could not find any reports on equal interventions with adults. Therefore, we decided to study a group of adults who had voluntary signed up to be assessed and treated for SMD and compare their results with a group of older children with an average age of 12 years (Niklasson et al., 2015).

KEY CONCEPT 5 **Sensorimotor disorder (SMD)**

SMD is a tentatively suggested complementary definition of the existing Developmental coordination disorder (DCD) diagnose. The difference is primarily the stressing of sensory causes such as vestibular impairment but also the importance of assessment and integration of remaining primary reflexes.

Training according to RB (Niklasson et al., 2007) is a process, which takes on average 3 years. During that time participants are doing exercises for about 15 min/day at home together with either their parents or their spouse. The method is a blend of five different approaches and the perspective includes the following notions:

- The deficit- or process-oriented approach (Wilson, 2004; Sugden, 2007; Blank et al., 2012; Smits-Engelsman et al., 2012) or the General Abilities Approach (GA) (Pless and Carlsson, 2000; Niklasson et al., 2009), which implies that deficits in underlying neurological structures needs to be remediated. A logic of RB as well as of GA is that persistent primary reflexes will become a hindrance for development of postural reactions and of gross motor milestones such as rolling, belly-crawling and creeping on hands and knees which in turn will affect the development of more complex motor skills.
- RB is also partly a Sensory Integration Approach (SI) (Pless and Carlsson, 2000; Niklasson et al., 2009) since the assumption that the development of motor skills is dependent on the individual's sensory integration ability. Another logic of RB is that vestibular stimulation plays a key role when it comes to the suppression of primary reflexes. During infancy the vestibular system is very responsive, reaching a peak between 6 and 12 months (e.g., Piontelli, 2015) a period which coincide with the development of belly-crawling, creeping on hands and knees and the child learning to walk (Capute and Accardo, 1991).
- A dynamic systems perspective on motor development stipulates that novel motor behavior emerge as a result of interactions between different subsystems. The nervous system is viewed as self-organizing and the process is arranged by the components themselves without any outside influence (e.g., Goldfield and Wolff, 2004). The terms emergence and self-organizing are in line with how we conceptualize the process of RB when primary reflexes are suppressed and novel gross motor patterns such as crawling and creeping on hands and knees emerge. However, we suggest that there is an outside agent, the ever-present gravitational force, which as the driving force, acts on the nervous system via the vestibular system (Hydén, 1961; Niklasson, 2012, 2013a; Adolph and Franchak, 2017). The process of RB is a “changing over time” and according to (van Geert, 2011), “*a dynamic system is a way to explain how the ‘next’ state of the system comes about as a result of its ‘preceding’ state.”*
- Bjorklund and Ellis (2014) argued that an evolutionary approach is of importance within the field of developmental psychology. Motor development organization obviously follows hierarchical principles where lower and automatic levels are inhibited by higher motor systems (Wiest, 2012) and the integrated layers of the brain are in accordance with hierarchical theory (e.g., MacLean, 1990) i.e., our evolutionary history (Wiest, 2012; Kirmayer and Crafa, 2014). MacLean's model on the evolution of the brain is also of importance for the understanding of emotions (LeDoux, 2004). In this way a hierarchical and neurodevelopmental concept has become a natural approach to sensorimotor training according to the method Retraining for Balance.
- In a previous qualitative study (Niklasson et al., 2010) the analysis indicated a strong connection between physiological exercises and psychological development through regressions and transformations. This was described through the conceptual Kinesthetic Vestibular Developmental Model (KVDM) (see below) which equals Piaget (1953) who wrote, “*The physiology of the organism furnishes a heredity mechanism which is already completely organized and virtually adapted but has never functioned. Psychology begins with the use of this mechanism.”* By “*heredity mechanism*” Piaget meant the infant's innate motor reflexes i.e., primary reflexes and further that in parallel with these reflexes fulfilling their aims, different psychological phenomena emerge (Cramer, 2006). A fundamental rationale of RB is that the developmental process could have been arrested but can be released through treatment (Gedo and Goldberg, 1973).

### Anamneses

In our recent study (Niklasson et al., 2015), prior to their first visit, the participants were interviewed on telephone by one of two therapists. After the conversation a decision was made as whether or not the individual should come for assessment. This verbal history taking is similar to the *Adult Developmental Co-ordination Disorders/Dyspraxia Checklist* (ADC) (Kirby et al., 2010) and to the *Movement ABC-2* checklist or the revised version of *the Developmental Coordination Disorder Questionnaire* (DCD-Q-R) (Wilson et al., 2009) for children. Regarding the children, Conners' *Teacher Rating Scale* (Conners, 1990; Janols and von Knorring, 1991; Niklasson et al., 2009) and Conners' *Parent Symptom Questionnaire* (Conners, 1973; Niklasson et al., 2009) were used before and after intervention. Adult participants, as well as the children's parents, were also asked to complete the questionnaire *Reasons for training* (Bergström et al., 1999) in order to indicate additional problems. Besides sensorimotor difficulties both groups reported attention problems, generalized anxiety, reading problems and sensitivity to stress. The similarity between the groups might be an indication that comorbidities do not wane with age and hints toward the importance of an early identification of sensorimotor problems in order to minimize additional problems later in life (Missiuna et al., 2015).

### Assessments

In contrast to the instruments *Movement ABC-2* (Henderson et al., 2007) and *Bruininks-Oseretsky Test of Motor Profiency-2* (Bruininks and Bruininks, 2005), which mainly assess the child's abilities on the functional level, the method *Retraining for Balance* uses instruments, which primarily evaluate motor patterns below the functional level (Niklasson and Niklasson, 2007a,b). The RB perspective is partly an answer to Wann (2007) who rhetorically wrote that although DCD is a heterogeneous diagnosis “*there must be common perceptuomotor subsystems that are poorly developed and refined*.” Previously, Sigmundsson (2003) stressed the importance of finding links or fundamental mechanisms between behavior and basic neural processing and argued for a process-oriented approach. We are well aware of the complexity of diagnosing developmental disorders (e.g., Dewey and Bottos, 2004; Flouris et al., 2005; Pennington, 2009; Zwicker et al., 2009) but when it comes to the visibility of sensory and motor difficulties and DCD one logic of RB has been that the lowest common denominator is the interaction between the vestibular system and primary reflexes (Niklasson et al., 2009, 2015). Both vestibular function and primary reflexes belong under the concept neurological soft signs (Ayd, 2000; American Psychiatric Association., 2013).

In our previous study (Niklasson et al., 2015) the concept sensorimotor disorder was tentatively suggested to label a diagnosis of DCD plus vestibular problems. Vestibular dysfunction has so far been overlooked within the framework of DCD but our studies (Niklasson et al., 2009, 2010, 2015) indicate the need for reconsideration. Recently the importance of a well-functioning vestibular system has been stressed in quite a few articles, connecting it to mental health (e.g., Gurvich et al., 2013), cognitive development (e.g., Weiner-Vacher et al., 2013) and a recent editorial (Besnard et al., 2015) declared that “*The sky's the limit for new ideas and developments in vestibular therapy (both pharmacological and physical devises).”*

The instruments used by the method Retraining for Balance are as follows:

- **RB-P**. *Retraining for Balance–Physiological Test* (RB-P) (Niklasson and Niklasson, 2007a; Niklasson et al., 2015) is a battery of 41 different subtests aiming at detecting aberrant primary reflexes (e.g., Capute et al., 1978; Blythe, 2009; Goddard Blythe, 2009) and determine the relation between primary reflexes, postural reactions, gross motor milestones, and sports-related gross motor skills (Capute and Accardo, 1991). Of special interest are primary reflexes (McPhillips et al., 2000; Zafeiriou, 2004) connected to the vestibular system i.e., the asymmetrical tonic neck reflex, the tonic labyrinthine reflex, the symmetrical tonic neck reflex and the Moro reflex. The primary reflexes, except the symmetrical tonic neck and the Moro reflexes are assessed in prone, supine, table position and standing position. There are different approaches to how to examine and evaluate neurological soft signs including primary reflexes in children (e.g., Touwen and Prechtl, 1970; Prechtl, 1977; Touwen, 1979; Vaivre-Douret et al., 2016) and adults (e.g., Rodnitzky, 1988). However, to our knowledge only RB uses vestibular related primary reflexes, postural reactions and gross motor skills in combination. A prerequisite for locomotion is the development of posture, which in turn is dependent on the integration of primary reflexes (Zafeiriou, 2004). During the first year of life, starting with the lifting of the head while in prone position the child is normally expected to defy the gravitational force via rolling, belly crawling, creeping on hands and knees and then further to the ability to walk. This developmental succession is of importance for RB. As shown in our previous study (Niklasson et al., 2015) both the younger group and the group of adults diverged from expected ability levels at first assessment. Of special interest is the ability to belly crawl, which we hold as an important transition between activities in prone position and the four feet position. Our experience is in line with both Adolph (2008) who wrote that only about 50% of the infants who later will creep on hands and knees also belly crawl and with Holt (1991) who showed that a skipping of belly crawl might lead to bottom shuffling. Not only will bottom shufflers walk later, they will also miss out valuable time in 4 feet position which should enable them to develop increasing strength in fingers, hands, neck and trunk and the ability to develop a cross pattern. A well-developed cross pattern is a prerequisite for the acquisition of sports-related gross motor skills. Our recent study (Niklasson et al., 2015) showed that even these skills were below expected levels in both groups before intervention.
- **RB-OB**. *Retraining for Balance-Orientation and Balance test* (RB-OB) (Niklasson and Niklasson, 2007b; Niklasson et al., 2009, 2015) is a group of tests aimed at detecting vestibular dysfunction and balance problems. Of special interest is the participants' reaction while being slowly rotated in a chair (e.g., Guyton, 1991; Barnett-Cowan, 2013) especially since it is common that children with sensorimotor problems prefer to move quickly.
- **RB-A**. *Retraining for Balance-Audiometric test* (RB-A) (Niklasson et al., 2009, 2015) is an auditory perceptual test aimed at detecting discrepancies between right- and left-ear dominance. A clinical diagnostic audiometer was used to measure ear preference according to a scale spanning from 0 to 200. Values below 100 indicated left ear dominance and values above 100 indicated right ear dominance. Speech sounds are supposed to be more rapidly processed through the right ear (Sininger and Cone-Wesson, 2004), which stresses the importance of a well-developed right-ear advantage (REA) early in life.
- **KVST**. *Keystone Visual Skills test* (Burman, 1977) is a visual skills test relating to vestibular function. The test has 14 subtests and uses 15 cards, which measures eye coordination, simultaneous perception, and effective acuity during resting accommodation at different distances as well as stereovision.

## Discussion

**Figure 1:**
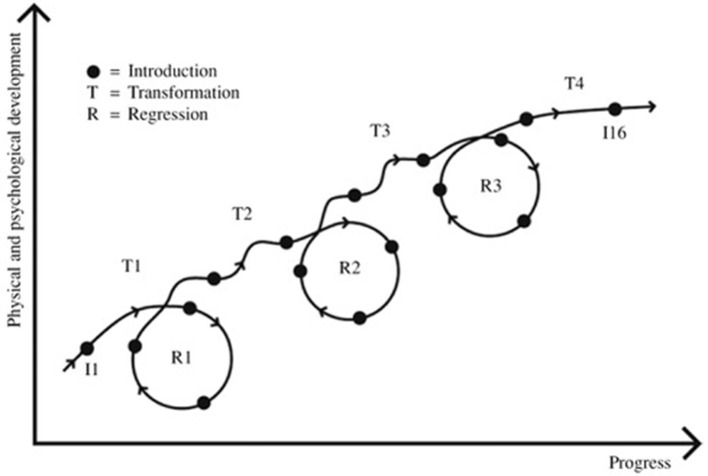
The participants needed close to 16 visits on average in order to complete the treatment in accordance with the sensorimotor therapy program Retraining of Balance. The visits (the introductions) are distributed along the curve above (I1–I16) and indicated as dots, During the treatment period three regression periods, here illustrated as circles (R1–R3), and four periods of transformation (T1–T4) were identified. (Reprinted from Social Behavior personality by kind permission. Originally Published 2010, 38(3), 335).

Our results have shown (Niklasson et al., 2010, 2015) that sensorimotor therapy (SMT) using primary reflex suppression and vestibular stimulation according to the method Retraining for Balance can be described in terms of a classical developmental curve containing plateaus, regressions (negative development), and transformations (positive development). It was through a qualitative analysis (Niklasson et al., 2010) with 8 children that the process, a change over time, became possible to describe through a conceptual Kinesthetic-Vestibular Developmental Model (KVDM) (Figure 1) thereby showing how the training (I) elicited temporary physical and psychological regressions (R) followed by transformations (T). The KVDM was validated in the same study through a comparison between the 8 children and 224 other children who had undergone the same treatment. In our recent study (Niklasson et al., 2015) the analysis showed no significant difference between children (mean age about 12 years) and adults (mean age about 35 years) regarding degree of alignment to the conceptual model. Neither were there any significant differences between the 2 age groups and a reference group compiled of 398 treated children (aged 4–17). Although children and adults showed the same patterns concerning periods of regressions and transformations, the regressions were often stronger among the younger participants.

The concept regression connotes a return to a hierarchically and developmentally lower level of psychological and/or physiological function (Wiest, 2012). We tentatively propose that a re-activation and integration of arrested primary reflexes together with vestibular stimulation causes regressions, which in turn are prerequisites for the release and emergence of both physiological and psychological transformations. We also speculate that the “*unidentified psychological barrier”* (Bergman and Norlander, 2005) mentioned above might be a result of vestibular dysfunction known to affect both emotions and body balance (Rajagopalan et al., 2017). Our results have indicated that sensorimotor problems in early life do affect not only physiological- but also psychological development. Thereby is the need for an early detection highlighted. One of us (Gillberg and Rasmussen, 2003) has previously argued for the urgent need of physical, neurological and neurodevelopmental examination of all children with learning and behavioral problems. As for now we find it appropriate to include children who show a sedentary behavior. Our results also indicated for the first time, as far as we know, that sensorimotor problems can be treated within all age groups.

The distinction between bottom-up and top-down approaches is too general and in need of revision (Berthoz and Petit, 2006). One of us has previously proposed (Niklasson, 2013b) an extension of the dynamical systems perspective on motor development (Goldfield and Wolff, 2004) through the inclusion of the vestibular system as a priming factor. Priming in the sense that repeated stimulus improves processing (Grill-Spector et al., 2006). Since priming can be viewed both as a basic perceptual process (bottom-up) and as an object-related process (top-down) it operates along the ascending as well as the descending loops in the nervous system (Rauss and Pourtois, 2013). We suggest, tentatively, that this is how sensorimotor therapy (SMT) (Niklasson et al., 2009, 2015) appears to work. In our conceptual model vestibular stimulation (perceptual priming) “ignites”/“nourish” the nervous system, assist the suppression of persistent primary reflexes thereby enhancing feedforward-feedback loops, making the nervous system more prone to object-related priming. In order to develop effective methods for assessment and intervention of DCD over the life span the importance of primary reflex suppression and vestibular stimulation as well as a combination of bottom-up and top-down approaches have to be considered.

## Ethics Statement

This Focused Review followed the ethical standards of the World Medical Association's Declaration of Helsinki concerning Ethical Principles of Medical Research involving Human Subjects and in accordance with the Swedish of Ethics.

## Author Contributions

All authors listed have made a substantial, direct and intellectual contribution to the work, and approved it for publication.

### Conflict of Interest Statement

The authors declare that the research was conducted in the absence of any commercial or financial relationships that could be construed as a potential conflict of interest.

## References

[B1] Adolph K. E. (2008). Motor and physical development: locomotion in Encyclopedia of Infant and Early Childhood Development, eds. Haith M. M. Benson J. B. (Amsterdam: Elsevier/Academic Press), 359–373.

[B2] Adolph K. E. Franchak J. M. (2017). The development of motor behavior. WIREs Cogn. Sci. 2017:e1430 10.1002/wcs.1430 PMC5182199 27906517

[B3] Ahonen T. Kooistra L. Viholainen H. Cantell M. (2004). Developmental motor learning disability. A neuropsychological approach, in Developmental Motor Disorders. A Neuropsychological Perspective, eds. Dewey D. Tupper D. E. (New York, NY: The Guilford Press), 265–290.

[B4] American Psychiatric Association (1994). American Psychiatric Association: Diagnostic and Statistical Manual of Mental Disorders, 4th Edn. Washington, DC: American Psychiatric Association.

[B5] American Psychiatric Association (2013). Diagnostic and Statistical Manual of Mental Disorders, 5th Edn. Arlington, VA: American Psychiatric Association.

[B6] Ayd F. J. Jr. (2000). Lexicon of Psychiatry, Neurology, and the Neurosciences. Philadelphia, PA: Lippincott Williams & Wilkins, 686.

[B7] Ayers A. J. (1972). Sensory Integration and Learning. Los Angeles, CA: Western Psychological Services.

[B8] Ayers A. J. (1989). Sensory Integration and Praxis Tests. Los Angeles, CA: Western Psychological Services.

[B9] Barnett A. L. (2014). Is there a ‘Movement Thermometer’ for developmental coordination disorder? Curr. Dev. Disord. Rep. 1, 132–139. 10.1007/s40474-014-0011-9

[B10] Barnett-Cowan M. (2013). Vestibular perception is slow: a review. Multisens. Res. 26, 387–403. 10.1163/22134808-00002421 24319930

[B11] Bergman A. Norlander T. (2005). Hay sacks anonymous”: living in the shadow of the unidentified. Psychological aspects of physical inactivity from a phenomenological perspective. Qual. Rep. 10, 795–816.

[B12] Bergström M. Niklasson M. Niklasson I. (1999). Reasons for Training. Mönsterås: Vestibularis.

[B13] Berthoz A. Petit J. L. (2006). The Physiology and Phenomenology of Action. Oxford: Oxford University Press.

[B14] Besnard S. Lopez C. Brandt T. Denise P. Smith P. F. (2015). Editorial: the vestibular system in cognitive and memory process in mammalians. Front. Integr. Neurosci. 9:55 10.3389/fnint.2015.00055 26617498 PMC4639622

[B15] Bjorklund D. F. Ellis B. J. (2014). Children, childhood, and development in evolutionary perspective. Dev. Rev. 34, 225–264. 10.1016/j.dr.2014.05.005

[B16] Blank R. Smits-Engelsman B. Polatajko H. Wilson P. (2012). European Academy of Childhood Disability (EACD): recommendations on the definition, diagnosis and intervention of developmental coordination disorder (long version). Dev. Med. Child. Neurol. 54, 54–93. 10.1111/j.1469-8749.2011.04171.x 22171930

[B17] Blythe P. (2009). Development of the INPP method - From theory to practice in Attention, Balance and Coordination. The ABC of Learning Success, ed Goddard Blythe S. (Chichester: Wiley-Blackwell), 311–323.

[B18] Bruininks R. H. Bruininks B. D. (2005). Bruininks-Oseretsky Test of Motor Proficiency, 2nd Edn. Winsor: NFER-Nelson.

[B19] Burman B. (1977). Keystone Visual Skills Test. Malmö: All-Optik and American Optical Co.

[B20] Caçola P. (2016). Physical and mental health of children with developmental coordination disorder. Front. Public Health 4:224. 10.3389/fpubh.2016.00224 27822464 PMC5075567

[B21] Capute A. J. Accardo P. J. (1991). Developmental Disabilities in Infancy and Childhood. Baltimore, MD: Paul Brooks.

[B22] Capute A. J. Accardo P. J. Vining E. P. G. Rubenstein J. E. Harryman S. (1978). Primitive Reflex Profile. Baltimore, MA: University Park Press. 10.1093/ptj/58.9.1061 684082

[B23] Conners C. K. (1973). Rating scales for use in drug studies. Psychopharmacol. Bull. 9, 24–29.

[B24] Conners C. K. (1990). Conners Rating Scales: Manual, Conners Teacher Rating Scales, Conners Parent Rating Scales: Instruments for use With Children and adolescents. North Tonawanda, NY: Multihealth Systems.

[B25] Cramer P. (2006). Protecting the Self. Defense Mechanisms in Action. New York, NY: Springer Verlag.

[B26] Dewey D. Bottos S. (2004). ”Neuroimaging of developmental motor disorders in Developmental Motor Disorders. A Neuropsychological Perspective, eds. Dewey D. Tupper D. E. (New York, NY: The Guilford Press), 26–43.

[B27] Dewey D. Creighton D. E. Heath J. A. Wilson B. N. Anseeuw-Deeks D. Crawford S. G.. (2011). Assessment of developmental coordination disorder in children born with extremly low birth weights. Dev. Neuropsychol. 36, 42–56. 10.1080/87565641.2011.540535 21253990

[B28] Feldenkrais M. (1988). Body and Mature Behaviour. Tel-Aviv: ALEF Ltd, 83–94.

[B29] Flouris A. D. Faught B. E. Hay J. Cairney J. (2005). Exploring the origins of developmental disorders. Dev. Med. Child Neurol. 47:436. 10.1017/S0012162205000848 15991861

[B30] Gedo J. E. Goldberg A. (1973). Models of the Mind. A Psychoanalytic Theory. Chicago, IL: The University of Chicago Press.

[B31] Gibbs J. Appleton J. Appleton R. (2007). Dyspraxia or developmental coordination disorder? Unravelling the enigma. Arch. Dis. Child 92, 534–539. 10.1136/adc.2005.088054 17515623 PMC2066137

[B32] Gillberg C. (2017). Developmental Coordination Disorder. Available online at: http://gillbergcentre.gu.se/english/research/diagnoses–methods-and-ongoing-studies-at-gnc/developmental-coordination-disorder–dcd-s

[B33] Gillberg C. Rasmussen P. (2003). To what extent are learning and behavioral problems brain related? Acta Psychiatr. Scand. 108, 81–82. 10.1034/j.1600-0447.2003.00128.x 12823163

[B34] Goddard Blythe S. (2009). Attention, Balance and Coordination. The abc of Learning Success. Chichester: Wiley-Blackwell.

[B35] Goddard Blythe S. (2014). Neuromotor Immaturity in Children and Adults. Chichester: Wiley-Blackwell.

[B36] Goldfield E. C. Wolff P. H. (2004). A dynamical systems perspective on infant action and its development, in Theories of Infant Development, eds Bremner G. Slater A. (Oxford: Blackwell Publishing Ltd), 3–29.

[B37] Gomez A. Sirigu A. (2015). Developmental coordination disorder:core sensori-motor deficits, neurobiology and etiology. Neuropsychologia 79(Pt B), 272–287. 10.1016/j.neuropsychologia.2015.09.032 26423663

[B38] Grill-Spector K. Henson R. Martin A. (2006). Repetition and the brain: neural models of stimulus-specific effects. Trends Cogn. Sci. 10, 14–23. 10.1016/j.tics.2005.11.006 16321563

[B39] Gurvich C. Maller J. J. Lithgow B. Haghgooie S. Kulkarni J. (2013). Vestibular insights into cognition and psychiatry. Brain Res. 1537, 244–259. 10.1016/j.brainres.2013.08.058 24012768

[B40] Guyton A. C. (1991). Basic Neuroscience: Anatomy and Physiology. Philadelphia, PA: Saunders.

[B41] Henderson S. Sugden D. Barnett A. (2007). Movement Assessment Battery for Children, 2nd Edn. Oxford: Pearson.

[B42] Hollenstein T. (2011). Twenty years of dynamic systems approaches to development: significant contributions, challenges, and future directions. Child Dev. Perspect. 5, 256–259. 10.1111/j.1750-8606.2011.00210.x

[B43] Holt K. S. (1991). Child Development: Diagnoses and Assessment. London: Butterworth-Heinemann.

[B44] Hydén H. (1961). Biochemical approaches of brain activity, in Man and Civilization: Control of the Mind, eds. Farger S. Wilson R. (New York, NY: McGraw-Hill), 18–41.

[B45] Janols L. O. von Knorring A. L. (1991). Är med medikamentell behandling motiverad vid hyperaktivitet hos barn? [Is stimulant drug action motivated when the child is hyperactive?]. Läkartidningen 88, 3057–3058. 1681156

[B46] Jorquera-Cabrera S. Romero-Ayuso D. Rodriguez-Gil G. Triviño-Juárez J. M. (2017). Assessment of sensory processing characteristics in children between 3 and 11 years old: a systematic review. Front. Pediatr. 5:57 10.3389/fped.2017.00057 28424762 PMC5371598

[B47] Kaplan B. J. Wilson B. N. Dewey D. Crawford S. G. (1998). DCD may not be a discrete disorder. Hum. Move. Sci. 17, 471–490. 10.1016/S0167-9457(98)00010-4

[B48] Kennedy J. Brown T. Stagnitti K. (2013). Top-down and bottom-up approaches to motor skill assessment of children: are child-report and parent-report perceptions predictive of children's performance-based assessment results? Scand. J. Occup. Ther. 20, 45–53. 10.3109/11038128.2012.693944 22646685

[B49] Kirby A. Edwards L. Sugden D. Rosenblum S. (2010). The development and standardization of the adult developmental co-ordination checklist (ADC). Res. Dev. Disabil. 31, 131–139. 10.1016/j.ridd.2009.08.010 19819107

[B50] Kirby A. Sugden D. A. (2007). Children with developmental coordination disorder. J. R. Soc. Med. 100, 182–186. 10.1177/014107680710011414 17404341 PMC1847727

[B51] Kirmayer L. J. Crafa D. (2014). What kind of psychiatry? Front. Hum. Neurosci. 8:435. 10.3389/fnhum.2014.00435 25071499 PMC4092362

[B52] Konicarova J. Bob P. (2012). Retained primitive reflexes and ADHD in children. Act. Nerv. Super. 54, 135–138. 10.1007/BF03379591

[B53] LeDoux J. (2004). The Emotional Brain. London: Phoenix.

[B54] Lin C. K. Kuo B. C. Wu H. M. (2014). Response to Niklasson's comment on Lin, et al. (2012): “The relation between postural movement and bilateral motor integration. Percept. Mot. Skills 119, 650–654. 10.2466/15.10.PMS.119c24z9 25310229

[B55] MacLean P. D. (1990). The Triune Brain in Evolution. Role in Paleocerebral Function. New York, NY: Plenum Press.

[B56] Mahoney G. Robinson C. Perales F. (2004). Early motor intervention: the need for new treatment paradigms. Infants Young Child. 17, 291–300. 10.1097/00001163-200410000-00003

[B57] Mailloux Z. (1990). An overview of sensory integration and praxis tests. Am. J. Occup. Ther. 44, 589–594. 10.5014/ajot.44.7.589 2386185

[B58] McPhillips M. Hepper P. G. Mulhem G. (2000). Effects of replicating primary-reflex movements on specific reading difficulties in children: a randomized, double blind, controlled trial. Lancet 355, 537–541. 10.1016/S0140-6736(99)02179-0 10683004

[B59] Missiuna C. Moll S. King G. Stewart D. Macdonald K. (2012). Life experiences of young adults who have coordination difficulties. Can. J. Occup. Ther. 75, 157–166. 10.1177/000841740807500307 18615927

[B60] Missiuna C. Polatajkajo H. J. Pollok N. (2015). Strategic management of children with developmental coordination disorder, in Developmental Coordination Disorder and its Consequences, ed Cairney J. (Toronto, ON: University of Toronto Press), 215–252.

[B61] Montoro A. P. P. N. Capistrano R. Ferrari E. P. da Silva Reis M. Luiz Cardoso F. Beltrame T. S. (2016). Concurrent validation of the MABC-2 and developmental coordination disorder questionnaire-BR. J. Hum. Growth Dev. 26, 74–80. 10.7322/jhgd.110421

[B62] Niklasson M. (2012). Could motor development be an emergent property of vestibular stimulation and primary reflex inhibition? A tentative approach to sensorimotor therapy, in Learning Disabilities, ed. W. Sittiprapaporn (Rijeka: InTech), 241–274.

[B63] Niklasson M. (2013a). The relation between postural movement and bilateral motor integration: comment on Lin, et al. (2012). Percept. Mot. Skills 117, 647–650. 10.2466/15.10.PMS.117x23z9 24611264

[B64] Niklasson M. (2013b). Sensorimotor Therapy: Assessing Quantitative and Qualitative Expressions of Physiological and Psychological Development in Children. Licentiate thesis, Faculty of Arts and Social Sciences, Karlstad University, Sweden.

[B65] Niklasson M. Niklasson I. (2007a). Retraining for Balance-Physiological Test Revised. Mönsterås: Vestibularis.

[B66] Niklasson M. Niklasson I. (2007b). Retraining for Balance-Orientation and Balance Test Revised. Mönsterås: Vestibularis.

[B67] Niklasson M. Niklasson I. Bergstöm M. (2007). Retraining for Balance Methods Revised. Mönsterås: Vestibularis.

[B68] Niklasson M. Niklasson I. Norlander T. (2009). Sensorimotor therapy: using stereotypic movements and vestibular stimulation to increase sensorimotor proficiency of children with attentional and motor difficulties. Percept. Mot. Skills 108, 643–669. 10.2466/pms.108.3.643-669 19725302

[B69] Niklasson M. Niklasson I. Norlander T. (2010). Sensorimotor therapy: physical and psychological regressions contribute to an improved kinesthetic and vestibular capacity in children and adolescents with motor difficulties and concentration problems. Soc. Behav. Pers. 38, 327–346. 10.2224/sbp.2010.38.3.327

[B70] Niklasson M. Rasmussen P. Niklasson I. Norlander T. (2015). Adults with sensorimotor disorders: enhanced physiological and psychological development following specific sensorimotor training. Front. Psychol. 6:480. 10.3389/fpsyg.2015.00480 25954233 PMC4406001

[B71] Norlander T. Moås L. Archer T. (2005). Noise and stress in primary and secondary school children: noise reduction and increased concentration ability through a short but regularly exercise and relaxation program. School Effectiv. School Improve. 16, 91–99. 10.1080/092434505000114173

[B72] Pannekoek L. Rigoli D. Piek J. P. Barrett N. C. Schoemaker M. (2012). The revised DCDQ: is it a suitable screening measure for motor difficulties in adolecents? Adapt. Phys. Activ. Q. 29, 81–97. 10.1123/apaq.29.1.81 22190054

[B73] Pennington B. F. (2009). Diagnosing Learning Disorders. A Neuropsychological Framework. New York, NY: The Guildford Press.

[B74] Peters J. M. Barnett A. L. Henderson S. E. (2001). Clumsiness, dyspraxia and developmental coordination disorder: how do health and educational professionals in the UK define the terms? Child Care Health Dev. 27, 399–412. 10.1046/j.1365-2214.2001.00217.x 11531913

[B75] Piaget J. (1953). The Origin of Intelligence in the Child. London: Routhledge and Kegan Paul LTD.

[B76] Piontelli A. (2015). Development of Normal Fetal Movements. The Last 15 Weeks of Gestation. Milan: Springer.

[B77] Pless M. Carlsson M. (2000). Effects of motor skill intervention on developmental coordination disorder: a meta-analysis. Adapt Phys. Act. Q. 17, 381–401. 10.1123/apaq.17.4.381 17091030

[B78] Poli E. Angrilli A. (2015). Great general startle reflex is associated with greater anxiety levels: a correlational study on 111 young women. Front. Behav. Neurosci. 9:10 10.3389/fnbeh.2015.00010 25705181 PMC4319476

[B79] Prechtl H. (1977). The Neurological Examination of the Full-Term Newborn Infant, 2nd Edn. Clinics in developmental medicine no. 63. London: William Heinemann Medical Books LTD.

[B80] Purcell C. Scott-Roberts S. Kirby A. (2015). Implications of DSM-5 for recognising adults with developmental coordination disorder (DCD). Br. J. Occup. Ther. 78, 295–302. 10.1177/0308022614565113

[B81] Rajagopalan A. Jinu K. V. Sailesh K. S. Mishra S. Reddy U. K. Mukkadan J. K. (2017). Understanding the links between vestibular and limbic systems regulating emotions. J. Nat. Sci. Biol. Med. 8, 11–15. 10.4103/0976-9668.198350 28250668 PMC5320810

[B82] Rasmussen P. Gillberg C. (2000). Natural outcome of ADHD with developmental coordination disorder at age 22 years: a controlled, longitudinal, community based study. J. Am. Acad. Child Adolesc. Psychiatry 39, 1424–1431. 10.1097/00004583-200011000-00017 11068898

[B83] Rauss K. Pourtois G. (2013). What is bottom-up and what is top-down in predictive coding? Front. Psychol. 4:276. 10.3389/fpsyg.2013.00276 23730295 PMC3656342

[B84] Rodnitzky R. L. (1988). Van Allen's Pictorial Manual of Neurological Tests. Chicago, IL: Year Book Medical Publishers, Inc.

[B85] Rousseau P. V. Matton F. Lecuyer R. Lahaye W. (2017). The moro reaction: more than a reflex, a ritualized behavior of nonverbal communication. Infant Behav. Dev. 46, 169–177. 10.1016/j.infbeh.2017.01.004 28222331

[B86] Schoemaker M. M. Wilson B. N. (2015). Screening for developmental coordination disorder in school-age children, in Developmental Coordination Disorder and its Consequences, ed Cairney J. (Toronto, ON: University of Toronto Press), 169–191.

[B87] Shumway-Cook A. Woollacott M. (1995). Motor Control. Theory and Practical Applications. Baltimore, MA: Williams and Wilkins.

[B88] Sigmundsson H. (2003). Perceptual deficits in clumsy children: inter- and intra- modal matching approach- a window into clumsy behavior. Neural Plast. 10, 27–38. 10.1155/NP.2003.27 14640305 PMC2565413

[B89] Sigmundsson H. Lorås H. Haga M. (2016). Assessment of motor competence across the life span: aspects of reliability and validity of a new test. SAGE Open 6, 1–10. 10.1177/2158244016633273

[B90] Sininger Y. S. Cone-Wesson B. (2004). Asymmetric cochlear processing mimics hemispheric specialization. Science 305:1581. 10.1126/science.1100646 15361617

[B91] Smits-Engelsman B. C. Blank R. van der Kaay A. C. Mosterd-van der Meijs R. Vlugt-van den Brand E. Polatajko H. J.. (2012). Efficacy of interventions to improve motor performance in children with developmental coordination disorder: a combined systematic review and meta-analysis. Dev. Med. Child. Neurol. 55, 229–237. 10.1111/dmcn.12008 23106530

[B92] Sugden D. (2007). Current approaches to intervention in children with developmental coordination disorder. Dev. Med. Child Neurol. 49, 467–471. 10.1111/j.1469-8749.2007.00467.x 17518935

[B93] Tal-Saban M. Zarka S. Grotto I. Ornoy A. Parush S. (2012). The functional profile of young adults with suspected developmental coordination disorder (DCD). Res. Dev. Disabil. 33, 2193–2202. 10.1016/j.ridd.2012.06.005 22789703

[B94] Thelen E. (2000). Motor development as foundation and future of developmental psychology. Int. J. Behav. Dev. 24, 385–397. 10.1080/016502500750037937

[B95] Thelen E. Smith L. B. (2006). Dynamic systems theory, in Handbook of Child Psychology, ed Lerner R. M. (Hoboken, NJ: John Wiley and Sons Inc.), 358–312.

[B96] Touwen B. C. L. (1979). Examination of the Child With Minor Neurological Dysfunction, 2nd Edn. Clinics in Developmental Medicine no. 71. London: William Heinemann Medical Books Ltd.

[B97] Touwen B. C. L. Prechtl H. F. R. (1970). The Neurological Examination of the Child With Minor Nervous Dysfunction. Clinics in developmental medicine no. 38. London: William Heinemann Medical Books Ltd.

[B98] Tupper D. E. Sondell S. K. (2004). Motor disorders and neuropsychological development. A historical appreciation, in Developmental Motor Disorders. A Neuropsychological Perspective, eds Dewey D. Tupper D. E. (New York, NY: The Guilford Press), 3–25.

[B99] Vaivre-Douret L. Lalanne C. Golse B. (2016). Developmental coordination disorder, an umbrella term for motor impairments in children: nature and co-morbid disorders. Front. Psychol. 7:502. 10.3389/fpsyg.2016.00502 27148114 PMC4832591

[B100] van Geert P. (2011). The contribution of complex dynamic systems to development. Child Dev. Perspect. 5, 273–278. 10.1111/j.1750-8606.2011.00197.x

[B101] Wann J. (2007). Current approaches to intervention in children with developmental coordination disorder. Dev. Med. Child Neurol. 49:405. 10.1111/j.1469-8749.2007.00405.x 17518922

[B102] Weiner-Vacher S. R. Hamilton D. A. Weiner S. I. (2013). Vestibular activity and cognitive development in children: perspectives. Front. Integr. Neurosci. 7:92 10.3389/fnint.2013.00092 24376403 PMC3858645

[B103] Wiest G. (2012). Neural and mental hierarchies. Front. Psychol. 3:516. 10.3389/fpsyg.2012.00516 23189066 PMC3505872

[B104] Wilson B. N. Crawford S. G. (2012). The Developmental Coordination Disorder Questionnaire 2007. Available online at: http://www.dcd.ca (Accessed March 23, 2017).

[B105] Wilson B. N. Crawford S. G. Green D. Roberts G. Aylott A. Kaplan B. J. (2009). Psychometric properties of the revised developmental coordination disorder questionnaire. Phys. Occup. Ther. Pediatr. 29, 182–202. 10.1080/01942630902784761 19401931

[B106] Wilson B. N. Kaplan B. J. Crawford S. G. Roberts G. (1998). The Development Coordination Disorder Questionnaire 2007. Calgary, AB: Alberta Children's Hospital Decision Support Research Team.

[B107] Wilson P. H. (2004). Practitioner review: approches to assessment and treatment of children with DCD: an evaluative review. J. Child Psychol. Psychiatry 46, 806–823. 10.1111/j.1469-7610.2005.01409.x 16033630

[B108] Zafeiriou D. I. (2004). Primitive reflexes and postural reactions in neurodevelopmental examination. Pediatr. Neurol. 31, 1–8. 10.1016/j.pediatrneurol.2004.01.012 15246484

[B109] Zwicker J. G. Missiuna C. Boyd L. A. (2009). Neural correlates of developmental coordination disorder: a review of hypotheses. J. Child Neurol. 24, 1273–1281. 10.1177/0883073809333537 19687388

